# Similarity maps and hierarchical clustering for annotating FT-IR spectral images

**DOI:** 10.1186/1471-2105-14-333

**Published:** 2013-11-20

**Authors:** Qiaoyong Zhong, Chen Yang, Frederik Großerüschkamp, Angela Kallenbach-Thieltges, Peter Serocka, Klaus Gerwert, Axel Mosig

**Affiliations:** 1 Department of Biophysics, CAS-MPG Partner Institute and Key Laboratory for Computational Biology, 320 Yueyang Road, 200031 Shanghai, China; 2 Department of Biophysics, Ruhr University Bochum, Universitätsstraße 150, 44801 Bochum, Germany

**Keywords:** Hierarchical clustering, Cluster validation, FT-IR microscopy, Raman microscopy, Image analysis, Similarity maps

## Abstract

**Background:**

Unsupervised segmentation of multi-spectral images plays an important role in annotating infrared microscopic images and is an essential step in label-free spectral histopathology. In this context, diverse clustering approaches have been utilized and evaluated in order to achieve segmentations of Fourier Transform Infrared (FT-IR) microscopic images that agree with histopathological characterization.

**Results:**

We introduce so-called *interactive similarity maps* as an alternative annotation strategy for annotating infrared microscopic images. We demonstrate that segmentations obtained from interactive similarity maps lead to similarly accurate segmentations as segmentations obtained from conventionally used hierarchical clustering approaches. In order to perform this comparison on quantitative grounds, we provide a scheme that allows to identify non-horizontal cuts in dendrograms. This yields a validation scheme for hierarchical clustering approaches commonly used in infrared microscopy.

**Conclusions:**

We demonstrate that interactive similarity maps may identify more accurate segmentations than hierarchical clustering based approaches, and thus are a viable and due to their interactive nature attractive alternative to hierarchical clustering. Our validation scheme furthermore shows that performance of hierarchical two-means is comparable to the traditionally used Ward’s clustering. As the former is much more efficient in time and memory, our results suggest another less resource demanding alternative for annotating large spectral images.

## Background

**Figure 1:**
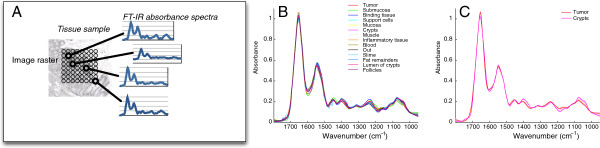
**Principle of FT-IR microscopy. (A)** At each pixel (indicated by circles), the infrared absorbance spectrum is measured, reflecting the biochemical status of the sample at the corresponding position. **(B)** Average spectra for different tissue components from a well established training dataset [3] exhibit relatively subtle differences on an absolute scale. **(C)** Mean spectra of crypts and tumor regions, with shaded areas around the mean spectra indicating standard deviation. Spectral variability within each class is small even in relation to the subtle differences between average spectra, so that differences between classes (here exemplified by crypts vs. tumor) remain distinguishable by classifiers.

In recent years, it has been well-established that label-free Fourier transform infrared (FT-IR) microscopy can resolve pathologically relevant information from histological tissue samples [1-3], as surveyed in [4]. Unveiling histopathologically relevant structures from localized absorbance spectra yielded by an FT-IR microscope, schematically illustrated in Figure 1, is typically achieved through a combination of unsupervised and supervised learning approaches [5,6]. First, certain number of spectrally measured tissue sections are being *annotated* based on pre-segmented spectral images, typically based on unsupervised clustering [6-8]. These annotations are then used to extract spectra as training data for supervised classifiers. Obviously, the quality of the annotation determines what tissue components can be resolved and how reliably they can be recognized by spectral classifiers. In this context, we introduce a novel interactive approach to annotation and quantitatively validate this approach in comparison to established annotation schemes. In particular, we provide novel algorithms to perform such quantitative comparison. As utilizing Raman [9] or CARS [10] microscopy often underlies the same workflow of data processing as FT-IR microscopy [11], the ideas discussed here may equally apply to these other types of label-free multispectral microscopy.

For annotating spectral images, at least two strategies are commonly employed. The first straightforward approach is to cluster all image spectra into a suitable number of *k* clusters. Each cluster is then identified with one index color, so that a pathologist may identify regions in the corresponding index color image with tissue components. As a second and typically complementary approach, the spectral image can be overlaid with a Hematoxylin and Eosin (H&E) stained image of the same tissue region. Pathologists can identify relevant tissue components in the H&E stained image, whose location patterns can be carried to the corresponding locations in the spectral image in order to extract spectra belonging to a certain tissue component. In practice, the accuracy in overlaying H&E stained images with FT-IR spectral images is limited, e.g. due to slight distortions of the tissue during the staining procedure. Also, there are limitations in identifying and marking up precise borders between certain tissue components, so that most approaches to FT-IR based spectral histopathology combine the two approaches [2,7,8]: A presegmentation of the spectral image is overlaid with the H&E stained image of the same sample, and then clusters in the spectral image are identified with tissue components based on their overlap with relevant regions in the H&E stained image, as identified by a pathologist. In general, the relation between clusters and tissue components is not one-to-one, but one tissue component may often be associated with several clusters. Thus, the number of clusters is usually chosen relatively large, so that the image is rather oversegmented. For obtaining presegmentations, hierarchical cluster analysis (HCA) [7] as well as *k*-means or fuzzy *c*-means [2,8] are common choices.

### Spectral image segmentation using similarity maps

As our main contribution, we introduce a novel interactive method for annotating FT-IR spectral images. Based on so-called similarity maps [12] and utilizing the concept of certain similarity measures between high dimensional vectors, annotations result from interactively choosing reference pixel spectra for the tissue components that can be identified in the tissue sample. By overlaying the interactive similarity maps (ISMs) with an H&E stained reference image, this allows to interactively take into account both spectral similarity and histopathological information from the stained image. This method is implemented in our so-called *Lasagne* software that has been originally proposed [13] and implemented [14] for multi-label fluorescence microscopy, while the present contribution adapts and quantitatively validates it for the use in vibrational microspectroscopy.

### Clustering and its validation for spectral image segmentation

In order to convincingly establish similarity maps as a suitable tool for infrared image annotation, it is essential to compare them to the currently predominant approach using clustering-based presegmentations. Clustering methods such as Ward’s method or *k*-means have been used extensively in infrared image segmentation and compared on a qualitative level for their suitability in vibrational microspectroscopy [7]. Yet, as has been noted prominently [15], “the validation of clustering is the most difficult and frustrating part of cluster analysis”. In fact, different applications and different clustering algorithms — in particular hierarchical ones — require different validation approaches. One application-specific consideration when employing and validating clustering in the context of infrared image annotation is that the correspondence between tissue components is not one-to-one, but rather one-to-many. Such considerations are rarely accounted for in existing validation procedures, and may be a reason that current comparisons of clustering approaches [7] are qualitative rather than quantitative. We address the lack of quantitative evaluation through introducing a validation scheme for hierarchical clustering based on so-called tree-assignments which were introduced in [16,17] in the context of tracking cells in live cell imaging. A very brief and preliminary validation of Ward’s clustering in FT-IR image segmentation was given in [3] and is fully detailed and systematically elaborated in the present work. In particular, we evaluate the suitability of hierarchical clustering approaches that are more efficient in terms of computational resources.

To identify a suitable validation scheme for unsupervised infrared image segmentation, it is important to consider in some more detail how clustering based segmentations are commonly used in this context. While not commonly described in detail, one typically attempts to choose a number of clusters that oversegments the image in a presegmentation. Then, the task of the human annotator is to identify each tissue component with one or several of these clusters. In some approaches, clusters may be extended or divided by navigating along the hierarchy of a given dendrogram. Using a ground truth segmentation as a reference, a validation scheme thus should aim to identify the best possible segmentation obtained from this workflow from a given clustering algorithm under realistic side assumptions.

One such side assumption is that there is a limit to the number of clusters that can be merged to represent one tissue component, which essentially represents a cognitive limit of a human annotator. Obviously, starting annotation from a crudely oversegmented image in general will allow more precise annotations. However, this comes at the cost of requiring the annotator to identify many small segments that merge into one tissue component. As there is no fixed limit to the degree of oversegmentation, we propose a validation scheme that takes the degree of oversegmentation into account as a parameter. We will refer to the degree of oversegmentation utilized during annotation as the *depth* of segmentation obtained from a dendrogram, and will introduce a validation scheme that allows to control segmentation depth through a parameter.

As surveyed in [18], a large diversity of validation measures for clustering algorithms has been proposed. Our main concern in this work is to validate clusters against a ground-truth reference segmentation, which is commonly referred to as *external validation*. While for external validation, one can principally rely on measures such as accuracy known from the validation for supervised classifiers, measures such as the Rand index and the Jaccard index [19,20], both in eventually normalized forms, are well established and commonly used. Furthermore, so-called *variation of information*[21] can be considered a well established information theoretic measure. However, these measures can only be applied to fixed segmentations, but not to dendrograms obtained from hierarchical clustering, and do not account for one-to-many relations between reference classes and clusters.

In infrared image annotation and other applications it is commonplace to use the dendrogram for obtaining a fixed partitioning into a certain number of classes. In this context, a straightforward and widely used approach horizontally cuts dendrograms into a fixed number of clusters [22]. In other words, given a dendrogram, one identifies edges $e_{1},\ldots ,e_{k}$ so that each *e*_*i*_ contains a point *v*_*i*_ that has same distance *δ* from the root for all *i*. Now, subtrees below these *k* edges define a partitioning into *k* classes. In general, however, there are numerous non-horizontal cuts supported by the same dendrogram that yield a different partitioning into the same number of clusters, which has been considered only recently in literature [23-25].

**Figure 2:**
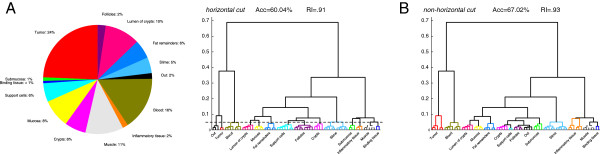
**Schematic overview of the cross-validation scheme for hierarchical clustering. (A) ** Composition of training data set, indicating an index color and proportion of spectra per class. **(B)** Dendrogram of the training data set and result of an optimal class assignment under a horizontal cut (indicated by dashed line in the left dendrogram) and an optimal tree assignment (right dendrogram) where each class is identified with the subtree colored according to its associated index color. Tree-assignment based segmentation not only achieves a much higher accuracy, but exhibits substantial differences in the assignment of several classes. The classes of crypts and submucosa are even identified as disjoint sets of spectra in both approaches, while substantial differences exist in the classes of tumour, inflammatory tissue, follicles, and support cells. The two segmentations indicate that even on well-curated training data, non-horizontal cuts in the dendrogram represent tissue classes much more reliably than horizontal cuts.

As illustrated in Figure 2, a segmentation based on a non-horizontal cut will generally reflect tissue components much better than a horizontal cut. Thus, validating different approaches to clustering in this context should take into account such non-horizontal cuts. Our contribution elaborates an approach that allows to systematically identify such non-horizontal cuts, yielding a corresponding validation scheme for hierarchical clustering. In particular, we utilize this scheme to quantitatively compare different hierarchical clustering approaches to interactive similarity maps. An important property of our validation scheme is that it can measure validity under different depths, i.e., different degrees of initial oversegmentation.

## Methods

### Interactive Similarity Maps (ISMs)

To introduce the concept of similarity maps following [12], let *F*(*x*,*y*) denote the FT-IR absorbance spectrum at position (*x*,*y*) in the spectral image. By choosing a reference spectrum *R* = *F*(*x*_*R*_,*y*_*R*_) at position *x*_*R*_,*y*_*R*_, one can measure the similarity between any position spectrum *F*(*x*,*y*) and the reference spectrum $R=(R_{1},\ldots ,R_{n})$ using a suitable measure of spectral similarity. Now, interpreting the spectral similarity at each position as an intensity, we obtain the similarity map *M* as an intensity image through

$$M_{R}(x,y)=\sigma_{R}(F(x,y)),$$

where *σ*_*R*_ measures the spectral similarity to the reference spectrum *R*. We follow the suggestions from [12] in using

(1) $$\begin{array}{l} \sigma_{R}(S):=\underset{i=1,\ldots ,n}{\prod }(1-|R_{i}-S_{i}{|}^{\alpha }) \end{array}$$

as our similarity function between reference spectrum $R=(R_{1},\ldots ,R_{n})$ and pixel spectrum $S=(S_{1},\ldots ,S_{n})$. Here, *α* is a non-negative real-valued parameter to adjust the sensitivity of the similarity measure. Note that Eq. 1 only makes sense if *R*_*i*_ and *S*_*i*_ range between 0 and 1. In practice, we achieve this by rescaling a dataset, and setting the minimum absorption occurring at any wavenumber in any spectrum to 0, and correspondingly setting the maximum absorption occurring in the dataset to 1.

Interpreting *σ*_*R*_(*S*) as a similarity measure between vectors *R* and *S*, it has been shown that Eq. (1) satisfies metric properties, which turns out to be a metric obtained by natural and systematic scheme to induce new metrics as products of other metrics [12]. A major advantage from a practical point of view is that Eq. (1) can be implemented on graphics hardware, so that the similarity map for all spectra from one image w.r.t. a given reference spectrum *R* can be computed within fractions of a second. This allows an interactive exploration of a spectral image by setting and moving the coordinate for the reference spectrum *R* with the mouse pointer.

In general, the product in Eq. (1) may vanish towards 0 rapidly if the difference is large for only a few features. The parameter *α* can be used to control this effect. Large values *α* lessen the tendency of the product to vanish towards 0 in datasets with heterogeneous features. Choosing a small *α* close to 0, on the other hand, can be used to amplify the tendency for the product to vanish when working on datasets with little variability. In practice, the parameter can adapted interactively, where common choices range between 1 and 2.

As demonstrated in Additional files 1, 2, 3, 4 and 5, an image can be annotated through similarity maps by interactively mouse-clicking one (or several) reference positions for each tissue component. This yields one reference spectrum *R* for each tissue component in the spectral image. Once a reference spectrum *R* is chosen, an intensity cutoff in the similarity map *M*_*R*_ can be set interactively. All positions (*x*,*y*) exceeding this threshold will be considered part of the same tissue component. If there are *K* different types of tissue components in the spectral image, annotation now reads as interactively identifying reference pixel spectra $R_{1},\ldots ,R_{K}$. In practice, as discussed in further detail in Results and discussion Section, some tissue components need to be represented by two rather than one reference spectrum. In case one position exceeds the threshold of several reference spectra, the position is assigned to the similarity map of highest intensity.

As an implementation, we utilized a version of the *Lasagne* software [13,14] adapted to the requirements of vibrational microspectroscopic data. A key feature of the *Lasagne* software is to perform computation of Eq. (1) on graphics hardware, so that the similarity maps *M* can be displayed in real-time. In our adapted version, the *Lasagne* software may also display overlays between the similarity maps and a reference image such as an H&E-staining image. In practice, annotation of an FT-IR microscopic dataset using the *Lasagne* software works as follows (see Additional files 1, 2, 3, 4 and 5): The annotator uploads both the FT-IR dataset and an H&E staining image of the same sample in the same coordinate system into the Lasagne software. Now, the spectrum at the current position of the mouse cursor in the image is interpreted as the reference spectrum to interactively display a similarity map. When moving the cursor to a suitable reference point, the similarity map may highlight a particular tissue compartment, which can be visually aligned by toggling between the similarity map and the H&E image. Once the correspondence between the similarity map and the tissue structure identifiable from the H&E image is visually well matched by varying the reference point, the annotator sets a suitable intensity threshold in the similarity map, so that the above-threshold positions can be used as training spectra for the corresponding tissue compartment. This may not only be done for one tissue compartment, but the annotator may set one (or even several) reference point for each tissue compartment that is identifiable in the given tissue sample. Each tissue compartment may be associated with an index color, so that the annotation can be interpreted as an index color image that resolves the tissue structure. In case of a conflicting position where several tissue compartments match the annotation, the position may be associated with the tissue compartment whose similarity map achieves the highest intensity.

In order to validate similarity maps for image annotation, we overlaid the spectral image with a reference segmentation obtained from a supervised classifier using a well-established set of training spectra [3]. Reference points for the different tissue components were set by a human operator, aiming to reproduce the reference segmentation as good as possible. While the annotation thus achieved may not be optimal in the sense that a different choice of reference points achieve a higher accuracy, it simulates a segmentation that may realistically achieved by a histopathologist.

### Hierarchical clustering

We employed two variants of hierarchical clustering. First, we hierarchically clustered spectra using Ward’s approach [26] based on two different distance measures. First, we employed the well-established and widely used correlation distance (i.e., one minus correlation coefficient) and, second, we used the power metric *d*_P_(*X*,*Y*) = 1 - *σ*_*X*_(*Y*) obtained using Eq. (1). As a further flavor of hierarchical clustering, we performed *hierarchical two-means*, i.e., recursively bipartitioning the dataset into two groups using two-means clustering in a top-down fashion. In each round of two-means clustering, the best subdivision among five repetitions on different random initialization was used for the next round of subdivision. For Ward’s clustering, we utilized the (parallelized) implementation provided by the *Statistics toolbox* of *Matlab* version 7.11. Hierarchical two-means clustering was implemented using *k*-means clustering provided by *Matlab*.

### Validation of hierarchical clustering

When performing hierarchical clustering on curated training data with training spectra for tissue components 1,…,K, a dendrogram obtained from an “ideal” hierarchical clustering would contain one vertex *v*_*i*_ for each i=1,…,K such that all spectra below *v*_*i*_ belong to class *i*. In order to measure to what degree a dendrogram *D*  obtained by Ward’s clustering achieves this criterion, we identify vertices $v_{1},\ldots ,v_{K}$ in *D* that approach this goal as far as possible. As detailed below, this can be achieved based on ideas behind so-called *tree-assignments* recently introduced in a different context in [16,17,27].

The main idea behind validating how well a given dendrogram reflects a given reference partitioning of a set of spectra is to utilize measures for comparing partitionings, such as accuracy or the popular Rand index (RI). Once such a measure is chosen, we determine a partitioning supported by the dendrogram that maximizes this measure. This approach is in line with the *VI-Cut* introduced in [24], which determines a partitioning that maximizes variation of information as an information theoretic measure for cluster validity. In terms of validation of infrared image segmentation and annotation, however, *VI-Cut* does not allow to control the depth of annotation. In fact, *VI-Cut* may in the end perform its validation on a segmentation derived from the dendrogram that realistically may not be recoverable by a human annotator.

#### ***Measures for comparing partitionings***

As our main validity measure for comparing partitionings, we use the *Rand index*[19], which is a well-established measure to compare two partitionings in the context of cluster validation [28]. The Rand index is defined for two partitionings  and $C^{\prime }$ that partition the set $\{1,\ldots ,n\}=C_{1}\cup \cdots C_{k}=C_{1}^{\prime }\cup \cdots C_{\ell }^{\prime }$. Following the notation from [28], the *Rand index* (RI) is based on the indicator function

$$e(i,j)=\left\{\begin{array}{cc} 1 & i,j\in C_{a}\;\text{for some}\;a\in \{1,\ldots ,k\}\\ 0 & \text{otherwise}. \end{array}\right)$$

and $e^{\prime }(i,j)$ correspondingly equal to one if *i* and *j* are in the same class in $C^{\prime }$ and 0 otherwise. We can now further define

$$\begin{array}{llll} n_{11} & = & \left|\{(i,j)|e(i,j)=e^{\prime }(i,j)=1\}\right| & \\ n_{00} & = & |\{(i,j)|e(i,j)=e^{\prime }(i,j)=0\}|, & \\ n_{01} & = & \left|\{(i,j)|e(i,j)=0,e^{\prime }(i,j)=1\}\right| & \\ n_{10} & = & |\{(i,j)|e(i,j)=1,e^{\prime }(i,j)=0\}|, & \end{array}$$

which finally yields the *Rand index *

$$R(C,C^{\prime })=\frac{2(n_{11}+n_{00})}{n(n-1)}.$$

We will also utilize the Mirkin metric, which as a close relative to the Rand index is defined as

$$ℳ(C,C^{\prime })=\overset{k}{\underset{i=1}{\sum }}|C_{i}{|}^{2}+\overset{\ell }{\underset{j=1}{\sum }}|C_{j}^{\prime }{|}^{2}-2\overset{k}{\underset{i=1}{\sum }}\overset{\ell }{\underset{j=1}{\sum }}m_{\text{ij}}^{2},$$

where $m_{\text{ij}}=|C_{i}\cap C_{j}^{\prime }|.$ Obviously, one can compute the Rand index easily from the Mirkin metric [28] using

(2) $$\begin{array}{l} R(C,C^{\prime })=1-\frac{ℳ(C,C^{\prime })}{n(n-1)}. \end{array}$$

#### ***Determining optimal partitionings supported by a dendrogram***

Given a dendrogram with *n* leaves and a reference partitioning $C^{\prime }$ that partitions the numbers {1,…,n}, we now aim to use the dendrogram to obtain a partitioning  that maximizes the Rand index between  and $C^{\prime }$. We allow to derive a partitioning from the dendrogram by assigning a class label to vertices in the dendrogram, so that all leaves below a labelled vertex *v* will belong to the assigned class. To prevent assignments of leaves to more than one class, no ancestor or descendant of an assigned vertex can be further assigned to a class.

Eq. (2) shows that indeed it is sufficient to minimize the Mirkin metric rather than maximizing the Rand index. Furthermore, the Mirkin metric is composed of 3 parts. Since $C^{\prime }$ is the reference partitioning, $\overset{\ell }{\underset{j=1}{\sum }}|C_{j}^{\prime }{|}^{2}$ is constant. Thus we only need to minimize the left 2 parts:

$$\begin{array}{lll} \;{ℳ}^{\prime } & =\overset{k}{\underset{i=1}{\sum }}|C_{i}{|}^{2}-2\overset{k}{\underset{i=1}{\sum }}\overset{\ell }{\underset{j=1}{\sum }}m_{\text{ij}}^{2}\; & \\ & =\overset{k}{\underset{i=1}{\sum }}\left(|C_{i}{|}^{2}-2\overset{\ell }{\underset{j=1}{\sum }}m_{\text{ij}}^{2}\right).\; & \end{array}$$

Let $w_{i}=|C_{i}{|}^{2}-2\overset{\ell }{\underset{j=1}{\sum }}m_{\text{ij}}^{2}$. Then

$$\;{ℳ}^{\prime }=\overset{k}{\underset{i=1}{\sum }}w_{i}.$$

Here *w*_*i*_ is the weight associated with class *C*_*i*_. |*C*_*i*_| is the number of leaves underneath vertex *v*_*i*_ and *m*_*ij*_ is the number of points shared by cluster *C*_*i*_ and $C_{j}^{\prime }$. Thus, the values *w*_*i*_ can be computed easily and quickly. The terminology introduce above suggests the following integer linear programming to identify an optimal partitioning:

(3) $$\begin{array}{ll} \text{min}\; & \overset{p}{\underset{i=1}{\sum }}w_{i}X_{i}\; \end{array}$$

(4) $$\begin{array}{ll} \text{s.t.}\; & \overset{k}{\underset{q=1}{\sum }}X_{v_{q}}=1\;\text{for each root-leaf path}\;(v_{1}\ldots v_{k})\; \end{array}$$

(5) $$\begin{array}{ll} & \overset{p}{\underset{i=1}{\sum }}X_{i}=Q\; \end{array}$$

(6) $$\begin{array}{ll} & X_{i}\in \{0,1\}\;\forall 1\leq i\leq p\; \end{array}$$

Here, *p* is the number of vertices in the dendrogram, *w*_*i*_ is the gained Mirkin metric if there is a cut at vertex *v*_*i*_ and *X*_*i*_ is a binary variable. *X*_*i*_ = 1 indicates that there is a cut at vertex *v*_*i*_. Finally, *Q* is the parameter that controls how many vertices may be assigned overall in the partitioning, thus controlling the depth of annotation: A small value of *Q* means the “annotator” has to choose large high vertices in the dendrogram to obtain the partitioning, a large value of *Q* means that the partitions can be merged from many small segments in lower parts of the dendrogram.

Once a tree-assignment has been obtained, it is useful to obtain a partitioning of the dataset where each partition is assigned one of the classes in the reference partitioning $C^{\prime }$. Such class assignment can be used to associate an accuracy of the segmentation , and in case of an image dataset can be used to produce an index color image. In order to obtain such class assignment, we follow a straightforward majority vote approach: Whenever a vertex *v*_*i*_ is active, i.e., *X*_*i*_ = 1, we need to associate the data points at the leaves below *v*_*i*_ with a class. By considering the labels of these *q* data points $x_{i,1},\ldots ,x_{i,q}$ in the reference partitioning $C^{\prime }$, we determine the label which occurs most often, and associate it with all leave data points $x_{i,1},\ldots ,x_{i,q}$.

Our tree-assignment implementation is based on the *Matlab* interface to version 5.5 of *lpsolve*. In order to limit the size of the ILP and avoid assignments to very low-level vertices, only the topmost 255 vertices in each dendrogram were allowed to be assigned. Note that this cutoff is far beyond what could be utilized in an HCA based annotation by histologists, as the resulting pre-segmented index color image involves at most 128 different index colors and thus appears highly fragmented. Thus, vertices located even lower in the dendrogram can be considered as un-identifiable in practice by an annotator. Meanwhile, we only need to compute the topmost 255 vertices in hierarchical two-means, which can reduce the running time even further.

If applied to a training dataset where each spectrum is assigned with a class label, the result of the tree-assignment reads as a re-classification of the training dataset. Thus, we can apply any validation measure used for measuring the quality of supervised classifiers. In particular, we can mimic validation schemes such as Monte-Carlo-type cross validation by repeatedly subsampling from the training dataset. In Results and discussion Section, we extensively utilize this idea to validate hierarchical clustering in comparison to both supervised classifiers and similarity-map based annotation.

### Datasets

For our computational studies, we utilized a colon tissue spectral dataset derived from [3]. The dataset consists of a training data set comprising 23,278 pixel spectra grouped into 14 classes of tissue components, along with three large spectral images displaying 854 × 502, 576 × 672 and 832 × 416 FT-IR pixel spectra of three tissue sections. The images will henceforth be referred to as *120514*, *88180* and *colon_p53_active*, respectively. The spatial resolution is 5.5 *μ*m/px. Following common practice in infrared image analysis, spectra exhibiting a weak signal or strong noise, e.g. resulting from holes or cracks in the tissue section or other artifacts, are discarded in a preprocessing step. This affects roughly 10% of all image spectra; for image *120514*, e.g., 8.24*%* of the image spectra are not considered for further analysis.

**Figure 3:**
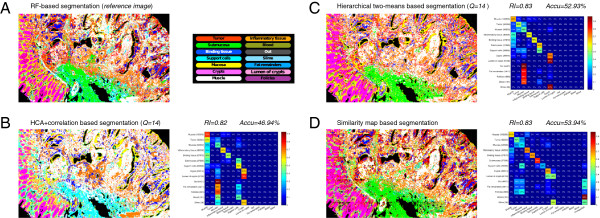
**Indexed spectral images and confusion matrices of image *120514 *. (A)**: Random-forest classified reference image. **(B-D)**: Segmentations and confusion matrices obtained by different annotation approaches. In the confusion matrices, the numbers beside the tissue names indicate class sizes, and the tissues are sorted by size in descending order. Ward’s clustering in combination with the power metric achieves an Rand index of 0.83 and accuracy of 53.35*%* (data not shown).

Based on the training dataset, a Random Forest classifier has been trained (for details, refer to [3]), yielding a segmented version of the spectral images that assigns one of the 14 trained classes to each pixel, see Figure 3(A). The training data set contains well-curated spectra and has been validated in detail in [3] by further experimental evidence using fluorescence microscopy. Furthermore, it was shown in [3] that the segmentations obtained from this supervised classifier resolve histopathologically relevant details such as the *lamina muscularis mucosae*. Following the general difficulty to obtain ground truth for biological image data [29], we used this fluorescence-validated and histopathologically well supported segmentation as a ground truth segmentation to quantitatively compare with segmentations obtained from similarity maps and hierarchical clustering algorithms. We may consider the cross-validation accuracy of the random forest of 94.92*%* on the training data as an estimate on the accuracy of our reference data.

## Results and discussion

To measure the performance of the classification and segmentation methods introduced here with other methods, we used the mean accuracy achieved in a Monte-Carlo type validation scheme whenever applicable.

### Validation of tree assignments

**Figure 4:**
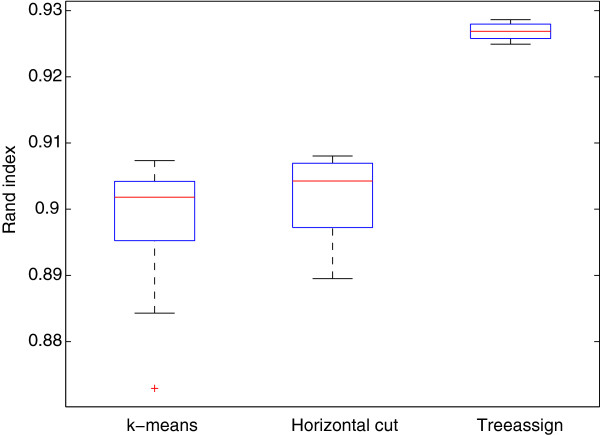
**Comparison of tree assignments, *k *-means and horizontal cut.** Clustering the training dataset into 14 classes using *k*-means (left) or Ward’s clustering using a horizontal cut (middle) leads to partitionings with a Rand index of around.9 with relatively high standard deviation. A partitioning obtained from Ward’s clustering using tree-assignments leads to a significantly higher Rand index (right). Note that the Rand index approaches 1 for datasets with many classes. Yet, the difference after Monte-Carlo type validation is clearly significant.

We compared segmentations obtained from tree assignments, *k*-means and horizontal cut (see Figure 4). In this case, horizontal cut performs slightly better than *k*-means. While non-horizontal cut using tree assignments gets much higher Rand index than the other two methods. Our results further confirmed the previous findings [7] in a systematic and quantitative way.

### Validation of similarity maps

We applied the *Lasagne* software to all the three spectral images, using the random forest classifications based on well-curated training data as reference segmentations. These RF-based reference segmentations were visually reconstructed as good as possible using the *Lasagne* software by a human operator. We allowed the operator to specify up to two reference pixel spectra per class. In the resulting segmentation of image *120514* (Figure 3), 376,718 (95.76*%*) out of the 393,378 non-background pixel spectra were assigned to one of the classes in the training dataset. The smallest five classes, namely *out*, *fat remainders*, *follicles*, *blood* and *slime* could not be properly identified as either too few spectra belong to this class (36 spectra for slime) or their location patterns were spectrally not unambiguously resolved by the *Lasagne* software. Yet, the resulting segmentation assigns 53.94*%* out of 393,378 pixel spectra to the correct class. This accuracy is higher than the accuracy achieved by either variant of HCA, where at most 53.35*%* of the spectra were assigned correctly by Ward’s clustering with the power metric. From the confusion matrices, we can see that both HCA based segmentations and similarity maps based segmentation perform better for big classes than for small classes. What is different is that for small classes that are difficult to identify, *Lasagne* rejects to assign any class label while HCA based methods make wrong assignments. In Figure 3(B), submucosa was totally mistakenly identified as either support cells or muscle, which is undesirable.

Figure 3 shows the RF-segmented reference image, tree assignments based segmentation and the *Lasagne*-reconstruction image for dataset *120514*. Corresponding results for the other two datasets *88180* and *colon_p53_active* are shown in Additional files 6 and 7. For dataset *88180*, the Rand index is equivalent between similarity maps and either variant of HCA (.75), while the accuracy is slightly higher for HCA based segmentations (≥ 59.39*%* for HCA vs. 56.23*%* for similarity maps). For dataset *colon_p53_active*, HCA accuracies are significantly higher (≥ 69.21*%* vs. 41.68*%*). Although HCA based segmentations received higher overall accuracies than similarity map based segmentation, many details of the tissue structure are lost. Due to the majority vote approach of class assignment subsequent to the tree-assignment based validation, they are more likely to mistakenly recognize small tissue classes as big tissue classes. This property may cause problems for samples containing unbalanced proportions of tissue classes. Furthermore, our validation is conservative in the sense that HCA is validated by a segmentation that algorithmically mimics the annotation that the “best possible annotator” could obtain from the given dendrogram, whereas the similarity map relies on a real human annotator to visually reproduce the ground truth segmentation.

### Validation of different hierarchical clustering approaches

**Figure 5:**
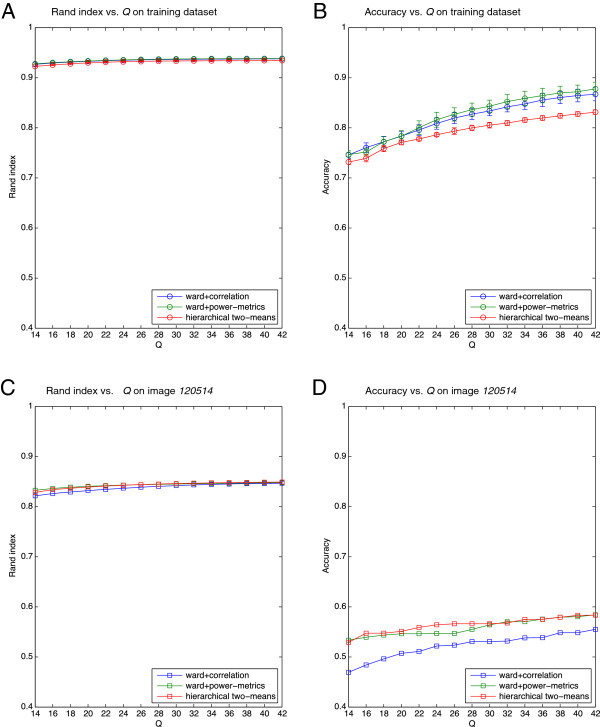
**Comparison of different hierarchical clustering approaches under varying the depth of segmentation *Q *. **Three hierarchical clustering schemes are evaluated in terms of Rand index and accuracy on both the training dataset **(A-B)** and image 120514 **(C-D)**. 10-fold Monte-Carlo cross validation is performed on the training dataset (the error bar indicates standard deviation).

We applied and evaluated tree assignments using different depths of segmentation Q=14,16,…,42 (see Figure 5 and Additional file 8). Both Rand index and accuracy increase with larger values of *Q* in essentially all cases. However, accuracy increases faster than the Rand index, which may be due to the relatively large number of 14 groups in our dataset, where the Rand index tends to approach 1. Hierarchical two-means performs worse than Ward’s method on training data, while comparable or even slightly better on image *120514*. In general, we may conclude that hierarchical two-means works well on image data, and using the power metric gives a slight, but not significant advantage over the established and widely used correlation distance on both image and training data. As to be expected, the accuracy achieved by unsupervised HCA using either distance measure is much smaller than the 94.92*%* accuracy obtained from a supervised random forest.

Beside validation measures, the running time required for obtaining clustering results is of high practical relevance. While not investigated in further detail, clustering roughly half a million image spectra using hierarchical two-means takes only few hours without parallelized computation, while Ward’s clustering consumes more than one week of computation time using up to 64 CPUs in parallel.

## Conclusions

We have introduced two novel concepts in the context of annotating FT-IR microspectroscopic images. First, we proposed a quantitative validation of hierarchical clustering schemes commonly employed during spectral image annotation. Second, we described and validated interactive similarity maps as an alternative to clustering-based image annotation.

### Similarity maps for vibrational microscopy image segmentation

Our contribution on interactive similarity maps suggests that there are viable alternatives to this “clustering paradigm”. As our findings suggest, annotations obtained using similarity maps may achieve similarly accurate as annotations based on hierarchical clustering. Compared to the costs of computing time and memory that are still significant even for the more efficient hierarchical two-means, similarity maps require no preprocessing beyond the commonly performed low-level normalization or baseline correction. Implemented on a GPU, re-computing the similarity map after an interactive change of a reference point can be done within fractions of a second even on large (>500,000 spectra) infrared images.

While visually identifying reference points is an intuitive concept addressing the histologist or pathologist not requiring any explicit computational expertise, this contribution provides a proof of concept based on quantitative validation. Establishing it to the level of a routine task for histologists or pathologists in larger scale studies is a perspective that should be encouraged by our positive quantitative validation of the approach. Both similarity-map based exploration and annotation and the concept of tree-assignments introduced here may be equally useful for Raman [9] and CARS [10] microscopy, which is worthwhile to explore in future contributions.

### Clustering in vibrational microscopy image segmentation

Along our contribution to quantitatively compare unsupervised infrared image segmentation strategies, we have provided a validation scheme for hierarchical clustering that matches the assumptions behind spectral image annotation, which turned out to be a non-trivial task in itself. As hierarchical clustering is arguably the most commonly used basis for infrared image annotation, this contribution is particularly important for systematically quantifying performance of different methods, rather than comparing by qualitative visual inspection. One of the immediate consequences we obtain is that the traditionally used Ward’s clustering may be substituted without significant loss of quality by hierarchical two-means for image segmentation. As the latter is much more time and memory efficient, this finding will make it much more practical to work with large spectral images. Being able to handle larger numbers of spectra without compromising in terms of accuracy becomes increasingly important in multispectral microscopy. In fact, the sizes of images keep growing with new generations of FT-IR microscopes and array detectors, or when working on confocally measured stacks of Raman or CARS images.

### Turning dendrograms into segmentations or partitionings

Finally, the idea of determining non-horizontal cuts in dendrograms and the cross-validation scheme based on this idea may be of further interest in infrared microscopy and beyond. Although not explored in this contribution, tree assignments also allow to compare two (or more) dendrograms by identifying an optimal set of classes supported by both dendrograms, rather than matching a fixed segmentation into one dendrogram. While this can achieved by relatively simple modifications of the integer linear programming and the weighting scheme provided here, exploration is left for future contributions.

## Abbreviations

FT-IR: Fourier transform infrared; H&E: Hematoxylin and Eosin; HCA: hierarchical clustering analysis; ISMs: interactive similarity maps; RF: random forest; ILP: integer linear programming; RI: Rand index.

## Competing interests

The authors declare that they have no competing interests.

## Authors’ contributions

QZ implemented clustering and validation algorithms, conducted computational experiments, drafted the manuscript, and participated in design of the study. CY participated in algorithm implementation. FG and AK prepared datasets. PS implemented software for interactive similarity maps. KG coordinated the study. AM conceived of the study, participated in its design, and drafted the manuscript. All authors read and approved the final manuscript.

## Supplementary Material

Additional file 1 **A video showing interactive annotation of a spectral image using *Lasagne *** **(part 1).** Click here for file

Additional file 2 **A video showing interactive annotation of a spectral image using *Lasagne *** **(part 2).** Click here for file

Additional file 3 **A video showing interactive annotation of a spectral image using *Lasagne *** **(part 3).** Click here for file

Additional file 4 **A video showing interactive annotation of a spectral image using *Lasagne *** **(part 4).** Click here for file

Additional file 5 **A video showing interactive annotation of a spectral image using *Lasagne *** **(part 5).** Click here for file

Additional file 6 **Indexed spectral images and confusion matrices of image *88180*.** Click here for file

Additional file 7 **Indexed spectral images and confusion matrices of image *colon_p53_active*.** Click here for file

Additional file 8 **Comparison of different HCA approaches on image *88180 *** **and *colon_p53_active*.** Click here for file
