# Supplementary material for: Similarity maps and hierarchical clustering for annotating FT-IR spectral images
Source: BMC Bioinformatics. 2013 Nov 20;14:333. doi: 10.1186/1471-2105-14-333 (PMC4225570; doi:10.1186/1471-2105-14-333)
Supplement: Additional file 6 — Indexed spectral images and confusion matrices of image 88180 . [file 1471-2105-14-333-S6.pdf]

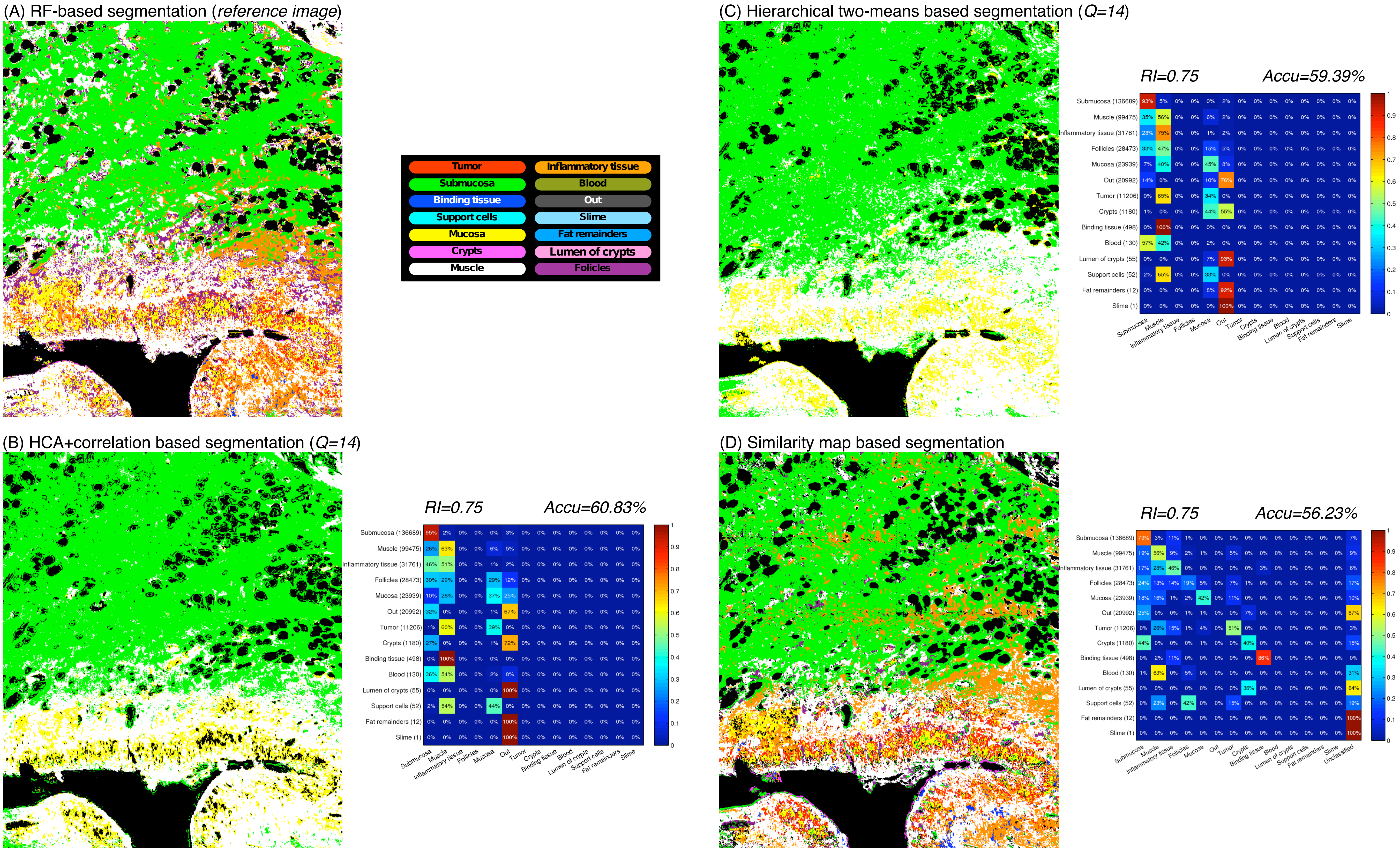

(A) RF-based segmentation (reference image)
Tumor
Inflammatory tissue
Submucosa
Blood
Binding tissue
Out
Support cells
Slime
Mucosa
Fat remainders
Crypts
Lumen of crypts
Muscle
Folicles
(B) HCA+correlation based segmentation (Q=14)
RI=0.75
Accu=60.83%
(C) Hierarchical two-means based segmentation (Q=14)
RI=0.75
Accu=59.39%
(D) Similarity map based segmentation
RI=0.75
Accu=56.23%
Submucosa (136689)
Muscle (99475)
Inflammatory tissue (31761)
Follicles (28473)
Mucosa (23939)
Out (20992)
Tumor (11206)
Crypts (1180)
Binding tissue (498)
Blood (130)
Lumen of crypts (55)
Support cells (52)
Fat remainders (12)
Slime (1)
Unclassified
