# Supplementary material for: Similarity maps and hierarchical clustering for annotating FT-IR spectral images
Source: BMC Bioinformatics. 2013 Nov 20;14:333. doi: 10.1186/1471-2105-14-333 (PMC4225570; doi:10.1186/1471-2105-14-333)
Supplement: Additional file 8 — Comparison of different HCA approaches on image 88180 and colon_p53_active . [file 1471-2105-14-333-S8.pdf]

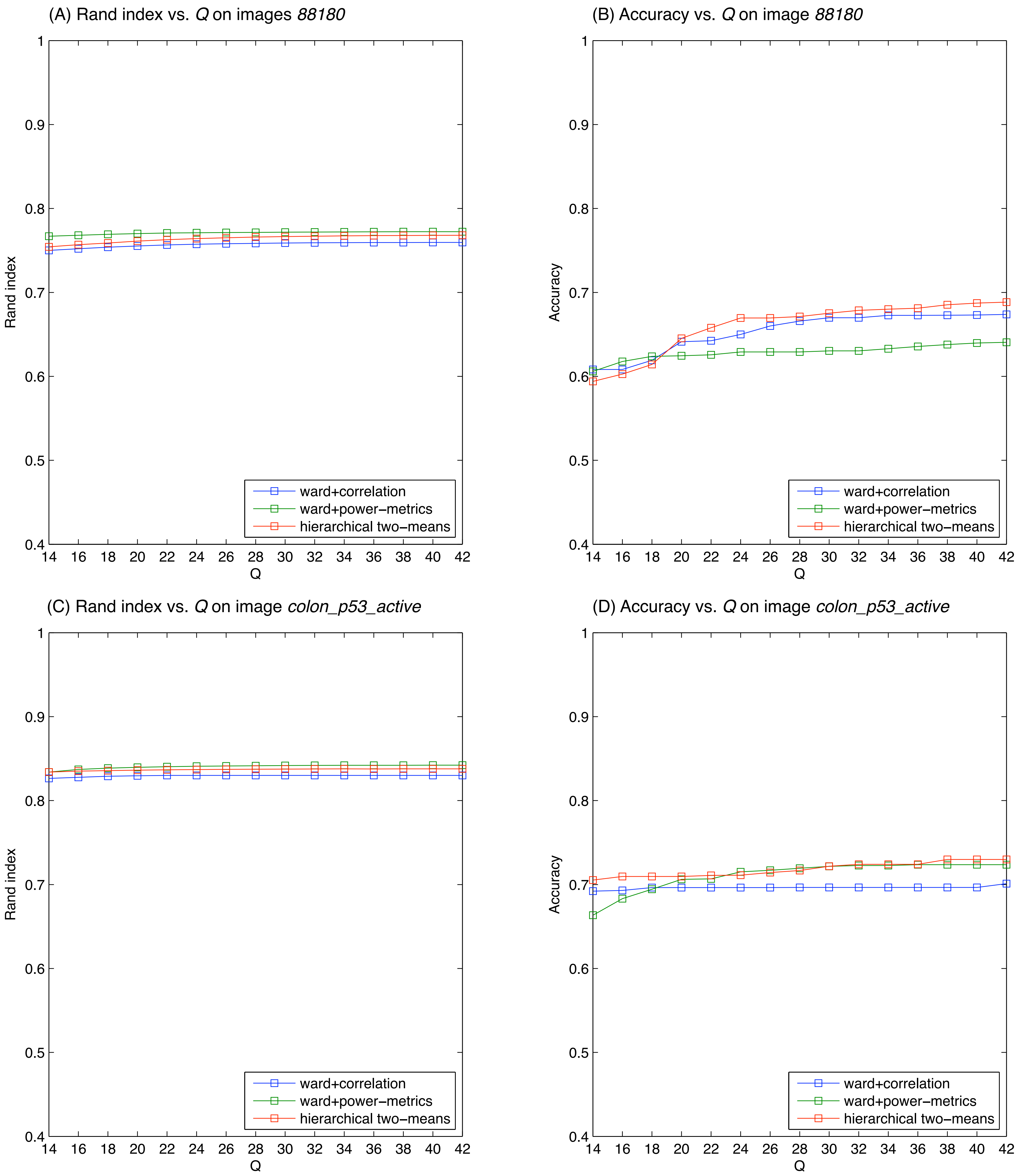
(A) Rand index vs. Q on images 88180
Rand index
Q
14 16 18 20 22 24 26 28 30 32 34 36 38 40 42
0.4 0.5 0.6 0.7 0.8 0.9 1
ward+correlation
ward+power−metrics
hierarchical two−means
(B) Accuracy vs. Q on image 88180
Accuracy
Q
14 16 18 20 22 24 26 28 30 32 34 36 38 40 42
0.4 0.5 0.6 0.7 0.8 0.9 1
ward+correlation
ward+power−metrics
hierarchical two−means
(C) Rand index vs. Q on image colon_p53_active
Rand index
Q
14 16 18 20 22 24 26 28 30 32 34 36 38 40 42
0.4 0.5 0.6 0.7 0.8 0.9 1
ward+correlation
ward+power−metrics
hierarchical two−means
(D) Accuracy vs. Q on image colon_p53_active
Accuracy
Q
14 16 18 20 22 24 26 28 30 32 34 36 38 40 42
0.4 0.5 0.6 0.7 0.8 0.9 1
ward+correlation
ward+power−metrics
hierarchical two−means
